# Impact of having a certified nurse specialist in critical care nursing as head nurse on ICU patient outcomes

**DOI:** 10.1371/journal.pone.0228458

**Published:** 2020-02-05

**Authors:** Tomohide Fukuda, Hironori Sakurai, Masanori Kashiwagi

**Affiliations:** 1 Faculty of Nursing, Kyoritsu Women’s University, Tokyo, Japan; 2 Department of Anesthesiology, National Hospital Organization Tokyo Medical Center, Tokyo, Japan; 3 Department of Anesthesiology, Tokyo Saiseikai Central Hospital, Tokyo, Japan; University of Oxford, UNITED KINGDOM

## Abstract

**Objectives:**

This study evaluated the impact of the presence of a certified nurse specialist in critical care (CNS) as ICU head nurse in an open ICU on clinical outcomes.

**Methods:**

The presence of a CNS as ICU head nurse was implemented in practice in April 2017. To evaluate the impact on patient outcomes before and after the implementation, patients were divided into two groups: before (April 2014 to March 2017; 1988 patients) and after (April 2017 to March 2019; 1664 patients). Patients’ demographic data were collected from the ICU database.

**Results:**

Multivariable logistic regression analysis revealed that the presence of a CNS as ICU head nurse was associated with lower ICU mortality (odds ratio (OR): 0.52, 95% CI: 0.36–0.73, *p* < .001) and fewer patients receiving mechanical ventilation in the ICU (OR: 0.20, 95% CI: 0.15–0.26, p < .001).

**Conclusion:**

CNSs are defined as one type of advanced practice nurses. Having a CNS as a head nurse in the ICU may have helped improve patient outcomes by leveraging these practical skills in nursing management.

## Introduction

Advanced practice nurses (APNs) are required to improve the quality of patient care and the health care systems of hospitals and regions. APNs are registered nurses who hold master’s degrees and have acquired the expert knowledge base, complex decision making skills, and clinical competencies necessary for expanded practice [1]. They are known by various titles such as clinical nurse specialists, nurse practitioners, nurse anesthetists, and nurse midwives.

Clinical nurse specialists, as expert clinical leaders, have obtained favorable outcomes in nursing interventions with patients in complicated situations and have demonstrated abilities in reducing medical costs [2–6]. In Japan, an APN who has a master’s degree is recognized as a Certified Nurse Specialist (CNS), a qualification that has been adopted based on the CNS designation in the United States.

Most CNSs work directly with patients or in a division of nursing education developing effective health care techniques based on clinical evidence, solving complex problems, and educating nurses [7–10]. In contrast, one-fifth of CNSs work as full-time nurse administrators such as head nurses, nursing vice-directors, or nursing directors. [11]

The role of nursing administrators, and especially head nurses, is overall administrative responsibility, which involves ensuring optimal quality nursing care in their units [12,13]. To improve the quality of care, head nurses set goals, monitor important outcomes, and evaluate initiatives.

Intensive care areas that are not managed by doctors specialized in intensive care (referred to as intensivists) are termed low intensity ICUs or open ICUs; particularly in these areas, facilitators are needed who have considerable clinical skills (e.g., deciding treatment policy, resolving various conflicts that arise during patient care). ICU head nurses often require not only management skills but also clinical skills to develop treatment policy and nursing care for patients with serious illnesses, cope with ethical problems, and convey the opinions of various clinical professionals as nursing advisors or caregivers. In some cases, these opinions and activities can change the treatment and nursing care a patient receives. Thus, a CNS acting in the head nurse’s role in critical care nursing may improve ICU patient outcomes by improving management’s role as head nurse and by directly enacting evidence-based care and treatment decision-making.

In nursing management science, there exists outcomes research regarding the working environment, patient safety, and leadership from the perspective of head nurses [14–18], but little work has focused on patient outcomes. Likewise, no work has been conducted on ICU patient outcomes in settings where a CNS is the head nurse. Therefore, this study evaluated the impact on patient outcomes of having a CNS as head nurse.

## Method

### Design

A retrospective cohort study was conducted among ICU patients in Japan over a five-year period. Data were collected from April 2014–March 2019. Results were compared between two groups: before and after a CNS was assigned as ICU head nurse.

### Target ICU

The targeted ICU is a general ICU with 10 beds that accepts hospitalized patients whose condition has rapidly changed and tertiary emergency patients, who are predominately postoperative patients. The director of the ICU was a cardiovascular surgeon until March 2016, but from April 2017 intensivists in anesthesiology were the main ICU management personnel. A system in which doctors provide treatment without receiving advice regarding intensive care was changed to a system called elective care consultation [19], in which the attending physician consults with intensivists when needed [20].

### CNS efforts

CNSs are required to perform six clinical roles: practice, coordination, ethical coordination, consultation, education, and research [10]. To form the basis of nursing practice to improve the quality of care in the ICU, practical nursing education was developed using guidelines and adult learning theory in accordance with treatment and nursing practices. Goals and progress on treatment, care, and rehabilitation are shared with physicians, intensivists, nurses, and other therapists, and are coordinated to improve treatment and care outcomes.

CNS-led multi-professional conferences were held for long-term ICU patients and cases with ethical issues: issues were noted, goals set, and future treatment and nursing in ICU was defined. When conflict that was difficult to solve occurred, the CNS encouraged consultation with the in-hospital ethics committee instead of seeking a solution on their own.

As a head nurse, before an elective care consultation for a complicated case or situation, the CNS discussed problems regarding the treatment plan and care based on patients’ backgrounds with the attending physician and staff nurses; the system was structured so that the intensivists could smoothly intervene. In addition, the ICU was effectively utilized. By referring to the medical judgment of intensivists, the necessity and extent of care were comprehensively examined, and patients who could leave ICU were identified. Other general management tasks of head nurses include setting ICU goals for nursing, managing staff, upgrading facilities, reducing medical costs, and decision making for the organization. In this way, the CNS fulfilled all of the roles of head nurse, and acted as a link between the attending physician, the intensivists in anesthesiology, the ICU nurses, and other medical staff.

### Data collection

The data of patients admitted to the ICU of a Japanese hospital between April 2014 and 2018 were collected from electronic medical records and the ICU ledger, namely: disease, patient age, medical department, scheduled or emergency admission, associated surgery, days in ICU, severity of patient’s condition, degree of medical and nursing needs in ICU, use of ventilator, hours of ventilation, and outcomes.

### Measurements

The severity of each patient’s condition and the extent of his or her required medical and nursing care were determined. The former was assessed using a scale that measures the degree of dependence on medical care for patients admitted to the ICU created by the Ministry of Health, Labor and Welfare of Japan (S1 and S2 Tables). The higher the score, the greater the degree of dependence on treatment and nursing care (severity). The score is divided into item A and item B. Item A scores medical dependency out of a maximum of 15 points in terms of presence or absence of electrocardiogram monitor, infusion pump, syringe pump, A-line, central venous catheter, and respirator attachment; use of transfusion and blood products and Swan-ganz catheter; and special treatment (intra-aortic balloon pumping [IABP], Continuous hemodiafiltration [CHDF], percutaneous cardiopulmonary support [PCPS], intracranial pressure measurement [ICP measurement], ventricular assist device [VAD], and extracorporeal membrane oxygenation [ECMO]). Item B scores the degree of dependence on nursing care out of a maximum of 12 points, in terms of the items turn over (unable to, can with assistance, can), transfer (cannot, needs assistance or observation, can), oral care (cannot perform, can perform), food intake (cannot feed self, requires assistance, can feed self), remove clothes (cannot, requires assistance, can), understand instructions regarding medical treatment (cannot understand, can understand), and dangerous behavior (yes, no). Evaluation criteria for each item apply nationwide and are evaluated daily for patients admitted to the ICU.

ICU nurses were responsible for scoring patients on this scale after completing training on how to do so via e-learning. After completing the training, all the nurses passed a computer-based examination.

### Data analysis

The period covered was divided into two intervals: before the ICU head nurse was a CNS (April 2014–March 2017) and after the ICU head nurse held this qualification (April 2017–March 2019). Differences between groups were compared using Fisher's exact test for categorical variables and the Mann-Whitney *U* test for continuous variables. The primary endpoint of this study was the ICU mortality rate, and the secondary endpoint was the number of ventilator-equipped patients in the ICU. Multiple logistic regression analysis was performed with dependent variables being the degree of severity and need for nursing care, and independent variables of presence of a CNS ICU head nurse, patient age, patient gender, and emergency versus planned admission to ICU. Significant differences were defined as *p* < .05. The free software Easy R (EZR) version 3.5.2 was used for analysis.

### Ethical considerations

The approval of the IRB of the Tokyo Saiseikai Central Hospital was obtained before the study commenced (No. 30–88). As this study did not involve any intervention, collecting informed consent from patients was judged unnecessary. The data obtained were password-protected and stored by the researchers. In consideration of privacy, patient names were not collected.

## Results

### Characteristics of patients

During the study period, data were collected from 3,652 people, with 1,988 in the first group (“before” group: ICU head nurse was not a CNS) and 1,664 in the second group (“after” group: ICU head nurse was a CNS). Patient characteristics are shown in Table 1. There was no difference between groups in age or gender. As intensivists began to take charge of ICU treatment management during the “after” period, a system was created that encouraged the surgical system to place high-risk patients into the ICU following an examination in anesthesiology at a preoperative consultation. Accordingly, postoperative ICU admissions increased in number. Additionally, ICU admissions increased for patients with gastrointestinal (*p* < .001) and respiratory (p < .001) issues. In contrast, establishment of a 10-bed emergency center ICU in the hospital after reorganization of the ward reduced the number of patients admitted to the ICU with cardiovascular disease (p < .001) from internal medicine and from emergency services (p < .001). As the number of patients admitted to the hospital increased, the severity of medical and nursing needs A score increased significantly (p < .001), although the B score decreased (p < .001). In addition, there were decreases in ICU admission days (p < .001), ICU mortality (p = .03), patients with ventilator (p = .003), and ventilator days (p < .001) due to the increase in planned surgeries. The A score increased, even though the number of patients requiring ventilation and the number of ventilation days decreased. This was due to an increase in patients treated with devices other than ventilators. In contrast, the B score decreased because the number of patients classified as exhibiting dangerous behavior decreased because the nursing team tried not to restrain individuals in the “after” period.

**Table 1:** Characteristics of patients.

	Before	After	*p* value
	*n* = 1,988	*n* = 1,664	
**Age, median (range)**	71 (9–105)	71 (19–106)	.12^a^
**Gender, number male (%)**	1411 (71.0)	1193 (71.7)	.65^b^
**Admission route**^b^			
Operating room	940 (47.3)	1141 (68.6)	< .001
Ward	308 (15.5)	198 (11.9)	.002
Emergency room	491 (24.7)	311 (18.7)	< .001
Other	249 (12.5)	14 (0.8)	< .001
**Emergency admission, number (%)**	1048 (52.7)	523 (31.4)	< .001^b^
**Emergency operation, number (%)**	140 (7.0)	153 (9.2)	.02^b^
**Diagnostic category, number (%)**^**b**^			
Cardiovascular	1044 (52.5)	582 (35.0)	< .001
Gastrointestinal	431 (21.7)	481 (28.9)	< .001
Respiratory	144 (7.2)	195 (11.7)	< .001
Neurological	64 (3.2)	85 (5.1)	.05
Hematologic	61 (3.1)	57 (3.4)	.60
Other	244 (12.3)	264 (15.9)	.002
**Severity of medical and nursing needs, A score (median, range)**	4 (1–15)	5 (1–15)	< .001^a^
**Severity of medical andnursing needs, B score (median, range)**	8 (2–19)	7 (1–12)	< .001^a^
**ICU stay, days (median, range)**	3 (1–60)	2 (1–52)	< .001^a^
**Deaths in ICU, number (%)**	130 (6.5)	70 (4.2)	< .001^a^
**Patients receiving mechanical ventilation (including NPPV**^c^**)**	753 (37.9)	550 (33.1)	.003^b^
**Mechanical ventilation, days (including NPPV**^c^**; median, range)**	3 (1–57)	3 (1–47)	.001^b^

^a^Mann-Whitney *U* test

^b^Fisher’s exact test

^c^Noninvasive positive-pressure ventilation.

In addition, there was no significant difference in the number of nurses (before: 24 staff nurses per month, range: 22–26; after: 25 staff nurses per month, range: 20–27; *p* = .075 by Mann-Whitney *U* test) and years of clinical experience (before: 5.3 years, range: 0–15 years; after: 5.1 years range: 0–14 years; *p* = .075 by Mann-Whitney *U* test) between the two groups, and no major changes in the medical devices used in the ICU during the study period.

### Relationship between presence of CNS ICU head nurse and patient outcomes

Table 2 shows the results of the multivariate logistic regression analysis of ICU mortality. The presence of a CNS as ICU head nurse was significantly associated with lower ICU mortality (OR: 0.52, 95% CI: 0.36–0.73, *p* < .001) and fewer ventilator-equipped patients in the ICU (OR: 0.20, 95% CI: 0.10–0.26, *p* < .001; Table 3).

**Table 2:** Association between CNS/Head Nurse Staffing and ICU mortality by multivariate logistic regression analysis.

Variable	OR (95% CI)	*p* value
CNS/head nurse staffing	0.52 (0.36–0.73)	< .001
Patient age	1.01 (0.99–1.02)	.314
Patient gender	1.23 (0.86–1.77)	.253
Emergency admission	4.39 (2.29–8.43)	.009
Non-operation	1.89 (1.04–3.45)	.037
Severity of medical and nursing needs, A score	1.31 (1.24–1.38)	< .001
Severity of medical and nursing needs, B score	1.28 (1.16–1.41)	< .001

**Table 3:** Association between CNS/Head Nurse Staffing and patients with mechanical ventilation by multivariate logistic regression analysis.

Variable	OR (95% CI)	*p* value
CNS/Head nurse staffing	0.20 (0.15–0.26)	< .001
Patient age	1.00 (0.99–1.01)	.794
Patient gender	1.07 (0.82–1.40)	.596
Emergency admission	1.75 (1.07–2.87)	.026
Non-operation	0.88 (0.53–1.45)	.617
Severity of medical and nursing needs, A score	2.54 (2.37–2.72)	< .001
Severity of medical and nursing needs, B score	1.20 (1.11–1.30)	< .001

## Discussion

A CNS conducts daily activities with a background of specialized medical and nursing knowledge and experience. The CNS contributes to patient outcomes through identifying problems and providing high-level practice, and through decision support and team building for dealing with difficult problems and patients in complex situations [21]. CNSs support stakeholders (i.e., patients and all ICU-related personnel) in addressing clinical problems and managing the care and treatment of patients. Further, CNSs fill an educational role for other nurses, which improves the quality of nursing care.

The head nurse manages ward policy, nursing staff, and finances to achieve ward goals so that the staff can engage in effective nursing practice. However, head nurses placed in a highly specialized ward may experience difficulties in setting outcomes and goals [13]. This is because treatments are complex and care has a therapeutic aspect that influences the outcome. In this study, a CNS acted as a head nurse; this individual communicated nursing policies while exchanging information with doctors, nursing staff, and other medical professionals, in order to improve the treatment received by patients. Notably, the presence of a CNS as head nurse was associated with a reduction in mortality among ICU patients and fewer patients requiring ventilators.

Previous research has shown that ICU management led by intensive care physicians is associated with improvements in mortality and the duration of hospitalization in Japanese and international contexts [20, 22]. Advanced practice nurses, including CNSs, have advanced nursing skills and clinical decision making experience in their area of expertise [23–25], which can facilitate positive outcomes by guiding the medical team to appropriate solutions [6, 26–28], especially for patients with complex and difficult problems in the ICU and general wards. In low-intensity ICUs or open ICUs, in which intensive care physicians do not determine treatment for various reasons, collaboration between physicians and the CNS responsible for advanced nursing practice improves treatment outcomes. It is essential that the CNS head nurse considers treatment in consultation with a physician, and in difficult cases, with intensivists, nurses, and other medical staff. The role of the CNS as a head nurse contributes to patient outcomes by predicting patient situations based on evidence and coordinating the use of nursing staff and medical device resources. The results of this study support the efficacy of ICU management performed by a CNS in low intensity and open ICUs. The fusion of clinical and management skills in CNS-qualified head nurses may contribute to positive ICU patient outcomes.

The main limitation of this study is that it was based in a single institution; thus, it is difficult to generalize these results to other institutions. Further, because many factors affect patients’ outcomes in a complex and mutually influential manner, it cannot be definitively concluded that the presence of a CNS as ICU head nurse directly affected patient outcomes. In addition, the scale used in this study reflected the severity of the patients’ conditions and the need for nursing care, but it is used only in Japan, and the correlation between severity as assessed by other methods, such as the APACHE severity of disease classification system and scales reflecting mortality, should be examined.

Research has been accumulating on CNS nursing interventions and CNS practice frameworks. Scientific analysis of the patient outcomes achieved by each CNS in their role, with a view to advanced practice nursing, will expand the role of the CNS.

## Conclusion

The presence of a CNS as ICU head nurse was associated with improved patient outcomes and fewer ventilated-equipped patients in the ICU. The fusion of advanced clinical skills and management skills may contribute to favorable patient outcomes in low intensity ICUs with a CNS as head nurse. In the future, additional qualitative and quantitative data should be collected to validate the utility and clinical value of CNS-qualified head nurses.

## Supporting information

S1 Table Severity, medical care, and nursing necessity in the ICU (Original edition in Japanese). (XLSX)

S2 Table Severity, medical care, and nursing necessity in the ICU (English version translated by authors). (XLSX)

## References

[1] ICN International NP/APN network. FAQ: What is a nurse practitioner/advanced practice nurse (NP/APN)? https://international.aanp.org/Home/FAQ (cited Nov 20,2019).

[2] Scott S, Denton L, Conway F, Kinley J. Managing health changes for people with a learning disability in a residential care home setting. Int J Palliat Nurs. 2019;25(11):531–540. 10.12968/ijpn.2019.25.11.531 31755831

[3] Tod AM, Redman J, McDonnell A, Borthwick D, White J. Lung cancer treatment rates and the role of the lung cancer nurse specialist: a qualitative study. BMJ Open. 2015;5(12):e008587 10.1136/bmjopen-2015-008587 26685023 PMC4691737

[4] Lawton K, Royals K, Carson-Chahhoud KV, Campbell F, Smith JB. Nurse-led versus doctor-led care for bronchiectasis. Cochrane Database Syst Rev. 2018;6:CD004359 10.1002/14651858.CD004359.pub2 29926473 PMC6513279

[5] Holtzer-Goor KM, Gaultney JG, van Houten P, Wagg AS, Huygens SA, Nielen MMJ, et al Cost-effectiveness of including a nurse specialist in the treatment of urinary incontinence in primary care in the Netherlands. PLoS One. 2015;10(10):e0138225 10.1371/journal.pone.0138225 26426124 PMC4591337

[6] Morita K, Matsui H, Yamana H, Fushimi K, Imamura T, Yasunaga H. Association between advanced practice nursing and 30-day mortality in mechanically ventilated critically ill patients: a retrospective cohort study. J Crit Care. 2017;41:209–215. 10.1016/j.jcrc.2017.05.025 28577478

[7] Quaal SJ. Clinical nurse specialist: role restructuring to advanced practice registered nurse. Crit Care Nurs Q. 1999;21(4):37–49. 10.1097/00002727-199902000-00007 10646431

[8] Semper J, Halvorson B, Hersh M, Torres C, Lillington L. Clinical nurse specialists guide staff nurses to promote practice accountability through peer review. Clin Nurse Spec. 2016;30(1):19–27. 10.1097/NUR.0000000000000157 26626744

[9] Miller S. The clinical nurse specialist: a way forward? J Adv Nurs. 1995;22(3):494–501. 10.1046/j.1365-2648.1995.22030494.x 7499617

[10] Anderson B. Challenges for the clinical nurse specialist in uro-oncology care. Br J Nurs. 2014;23(10 sup):S18–S22. 10.12968/bjon.2014.23.Sup10.S18 24851805

[11] Japan Nursing Association. List of registered persons by job position of hospital workers. December 2017 [in Japanese]. http://nintei.nurse.or.jp/nursing/wp-content/uploads/2018/03/3_byouinnkinnmusya_bunnyabetsu_syokuibetsu.xlsx (cited 2019 Apr 30).

[12] Warshawsky N, Cramer E. Describing nurse manager role preparation and competency: findings from a national study. J Nurs Adm. 2019;49(5):249–255. 10.1097/NNA.0000000000000746 30973429

[13] McCallin AM, Frankson C. The role of the charge nurse manager: a descriptive exploratory study. J Nurs Manag. 2010;18(3):319–325. 10.1111/j.1365-2834.2010.01067.x 20546472

[14] Halter M, Boiko O, Pelone F, Beighton C, Harris R, Gale J, et al The determinants and consequences of adult nursing staff turnover: a systematic review of systematic reviews. BMC Health Serv Res. 2017;17:824 10.1186/s12913-017-2707-0 29246221 PMC5732502

[15] Armstrong SJ, Rispel LC, Penn-Kekana L. The activities of hospital nursing unit managers and quality of patient care in South African hospitals: a paradox? Glob Health Action. 2015;8:26243 10.3402/gha.v8.26243 25971397 PMC4430688

[16] Basu S, Harris A, Mason S, Norman J. A longitudinal assessment of occupational stress in emergency department nursing staff. J Nurs Manag. 2019; in press. 10.1111/jonm.12910 31756010

[17] Abdelhafiz IM, Alloubani AM, Almatari M. Impact of leadership styles adopted by head nurses on job satisfaction: a comparative study between governmental and private hospitals in Jordan. J Nurs Manag. 2016;24(3):384–392. 10.1111/jonm.12333 26310389

[18] Suominen T, Savikko N, Puukka P, Doran DI, Leino-Kilpi H. Work empowerment as experienced by head nurses. J Nurs Manag. 2005;13(2):147–153. 10.1111/j.1365-2934.2004.00523.x 15720484

[19] Cooke L, Gemmill R, Grant M. APN core competencies: a framework for developing and testing an APN discharge intervention. Clin Nurse Spec. 2008;22(5):218–225. 10.1097/01.NUR.0000325366.15927.2d 18753879 PMC3097039

[20] Saito K, Yasui Y, Uchino S, Endo A, Iwai K, Kase Y, et al Physician staffing in a Japanese intensive care unit. Nihon Shuchu Chiryo Igakukai Zasshi. 2014;21:195–198. [in Japanese]

[21] Fulton JS, Mayo A, Walker J, Urden DL. Description of work process used by clinical nurse specialist to improve patient outcomes. Nurs Outlook. 2019;67(5):511–522. 10.1016/j.outlook.2019.03.001 31030905

[22] Pronovost PJ, Angus DC, Dorman T, Robinson KA, Dremsizov TT, Young TL. Physician staffing patterns and clinical outcomes in critically ill patients and clinical: a systematic review. JAMA 2002;288:2151–2162. 10.1001/jama.288.17.2151 12413375

[23] Riordan F, McHugh MS, Murphy K, Barett J, Kearney MP. The role of nurse specialist in the delivery of integrated diabetes care: a cross-sectional survey of diabetes nurse specialist services. BMJ Open. 2017;7:e015049, 10.1136/bmjopen-2016-015049 28801394 PMC5724109

[24] Roberts LA, Potts WWH, Stevens C, Lally P, Smith P, Fisher A. Cancer specialist nurses’ perspective of physical activity promotion and the potential role of physical activity apps in cancer care. J of Cancer Nurs. 2019; in press. 10.1007/s11764-019-00801-w. PMC6828618 31475306

[25] Nozue K, Usami S, Fukuda N, Takehara T, Ishii M, Fukushima Y, et al Randomized controlled study evaluating the intervention effect of certified nurse specialist in psychiatric mental health nursing on depressive cancer patients. J Jpn Acad Nurs Sci. 2016;36:147–155 [in Japanese].

[26] Crosbie R, Cairney J, Calder N. The tracheostomy clinical nurse specialist: an essential member of the multidisciplinary team. J Laryngol Otol. 2014;128(2):171–173. 10.1017/S0022215114000024 24480022

[27] Coen J, Curry K. Improving heart failure outcomes: the role of the clinical nurse specialist. Crit Care Nurs Q. 2016;39(4):335–344. 10.1097/CNQ.0000000000000127 27575796

[28] Kirk AP, McGlinsey A, Beckett A, Rudd P, Arbour R. Restraint reduction, restraint elimination, and best practice: role of the clinical nurse specialist in patient safety. Clin Nurse Spec. 2015;29(6):321–328. 10.1097/NUR.0000000000000163 26444510

